# Treatment of Hepatic Artery Stenosis in Liver Transplant Patients Using Drug-Eluting versus Bare-Metal Stents

**DOI:** 10.3390/jcm10030380

**Published:** 2021-01-20

**Authors:** Sailendra Naidu, Sadeer Alzubaidi, Grace Knuttinen, Indravadan Patel, Andrew Fleck, John Sweeney, Bashar Aqel, Brandon Larsen, Matthew Buras, Michael Golafshar, Rahmi Oklu

**Affiliations:** 1 Division of Vascular & Interventional Radiology, Mayo Clinic Arizona, Phoenix, AZ 85054, USA; Alzubaidi.Sadeer@mayo.edu (S.A.); knuttinen.grace@mayo.edu (G.K.); Patel.Indravadan@mayo.edu (I.P.); fleck.drew@mayo.edu (A.F.); oklu.rahmi@mayo.edu (R.O.); 2 Division of Cardiovascular Diseases, Mayo Clinic Arizona, Phoenix, AZ 85054, USA; sweeney.john3@mayo.edu; 3 Division of Gastroenterology and Hepatology, Mayo Clinic Arizona, Phoenix, AZ 85054, USA; Aqel.Bashar@mayo.edu; 4 Division of Anatomic Pathology, Mayo Clinic Arizona, Phoenix, AZ 85054, USA; Larsen.Brandon@mayo.edu; 5 Division of Biostatistics, Mayo Clinic Arizona, Phoenix, AZ 85054, USA; Buras.Matthew@mayo.edu (M.B.); Golafshar.Michael@mayo.edu (M.G.)

**Keywords:** arterial occlusive diseases/therapy, hepatic artery/physiopathology, liver transplantation/adverse effects, stents, vascular patency

## Abstract

Hepatic artery stenosis after liver transplant is often treated with endovascular stent placement. Our institution has adopted use of drug-eluting stents, particularly in small-caliber arteries. We aimed to compare patency rates of drug-eluting stents vs. traditional bare-metal stents. This was a single-institution, retrospective study of liver transplant hepatic artery stenosis treated with stents. Primary patency was defined as time from stent placement to resistive index on Doppler ultrasonography (<0.5), hepatic artery thrombosis, or any intervention including surgery. Fifty-two patients were treated with stents (31 men; mean age, 57 years): 15, drug-eluting stents; 37, bare-metal stents. Mean arterial diameters were 4.1 mm and 5.1 mm, respectively. Technical success was 100% (52/52). At 6 months, 1, 2, and 3 years, primary patency for drug-eluting stents was 80%, 71%, 71%, and 71%; bare-metal stents: 76%, 65%, 53%, and 46% (*p* = 0.41). Primary patency for small-caliber arteries (3.5–4.5 mm) with drug-eluting stents was 93%, 75%, 75%, and 75%; bare-metal stents: 60%, 60%, 50%, and 38% (*p* = 0.19). Overall survival was 100%, 100%, 94%, and 91%. Graft survival was 100%, 98%, 96%, and 90%. Stenting for hepatic artery stenosis was safe and effective. While not statistically significant, patency improved with drug-eluting stents compared with bare-metal stents, especially in arteries < 4.5 mm in diameter. Drug-eluting stents can be considered for liver transplant hepatic artery stenosis, particularly in small-caliber arteries.

## 1. Introduction

Hepatic artery stenosis (HAS) occurs in 4% to 11% of patients after a liver transplant [1,2]. If untreated, HAS can progress to hepatic artery thrombosis in up to 65% of patients [3]. Hepatic artery thrombosis can be associated with substantial morbidity, graft dysfunction, and potential for re-transplant [4].

When HAS is diagnosed, treatment options include surgical revision or endovascular therapies, such as balloon angioplasty, stent placement, or both. Endovascular therapy is the usual approach, unless a patient has an anatomical contraindication. Over the past decade, stent placement has been favored for HAS over primary angioplasty, given the lower rates of re-intervention and improved patency rates associated with stents [5,6].

However, stents can develop restenosis from intimal hyperplasia which may require an additional intervention such as angioplasty or additional stent placement [7]. In particular, small-diameter stents are prone to in-stent restenosis [8]. Furthermore, transplant arteriopathy may cause arteries to be particularly prone to restenosis [9]. Compared with bare-metal stents (BMS), drug-eluting stents (DES), which decrease cellular proliferation and intimal hyperplasia, have shown superior patency in the treatment of coronary artery disease [10]. DES have also been used to treat transplant renal artery stenosis (TRAS) [11,12].

The utility of DES versus bare metal stents in transplant liver hepatic artery stenosis has not been previously described. The main objective of this study was to compare the primary patency rates of DES and BMS in liver transplant patients with HAS. The secondary objectives were to determine technical success, complications, and overall patient and graft survival rates.

## 2. Materials and Methods

We identified patients for the study through electronic health records and included recipients of liver transplants who were diagnosed with HAS and treated with BMS or DES from 7 February 2003 through 20 September 2018 at our tertiary-care center. We excluded patients who underwent diagnostic angiography only, angioplasty only, or surgical revision. Patients with hepatic artery thrombosis requiring thrombectomy or thrombolysis were also excluded.

We recorded pre-procedural clinical and demographic data including interval time from liver transplant, diagnostic studies leading to the diagnosis of HAS, and serum laboratory values. Doppler ultrasonography was the most common test used to diagnose HAS. Patients with a resistive index (RI) less than 0.5 in either the left or right hepatic artery were considered to have substantial HAS, warranting treatment. Patients with abnormal ultrasonography findings underwent computed tomography angiography (CTA) for further evaluation, determining the feasibility of endovascular intervention, and procedural planning (femoral, brachial, or radial access).

Digital subtraction angiography (DSA) was performed with a 5-Fr catheter placed in the celiac trunk. Either a 0.014″ or 0.035″ wire was advanced into the hepatic artery, and a guide catheter or sheath was advanced into the celiac trunk or proximal common hepatic artery. DSA imaging was performed at optimal angulation, based on the planning CTA. Heparin (3000–5000 U) was administered. Nitroglycerin was administered as needed intra-arterially to prevent vasospasm. The stenotic area was crossed with a wire and a 3-mm balloon used to dilate the stenosis, after which a stent was placed across the narrowing. The stent length was chosen to avoid any substantial residual narrowing in the artery. Stents could be dilated up to 1 mm from their nominal diameters. In general, DES were used for small-diameter vessels (<5 mm); however, stent choice was up to the operator. Specific DES used were Xience everolimus-eluting stent (Abbott Vascular, Santa Clara, CA, USA), Promus Premier everolimus-eluting stent (Boston Scientific, Natick, MA, USA) Synergy everolimus-eluting stent (Boston Scientific, Natick, MA, USA), Resolute Onyx zotarolimus-eluting stent (Medtronic, Minneapolis, MN, USA) and Cypher sirolimus-eluting stent (Cordis Corp, Johnson & Johnson, Warren, NJ, USA). BMS included Liberté and Veriflex (Boston Scientific, Natick, MA, USA), Herculink Elite (Abbott Vascular, Santa Clara, CA, USA) Xpert (Abbott Vascular, Santa Clara, CA, USA), Express LD (Boston Scientific, Natick, MA, USA), and Protégé (Medtronic, Minneapolis, MN, USA). DSA imaging was used to confirm patency, no residual stenosis, and restored flow to the liver.

Immediately after the procedure, all patients received loading doses of aspirin (325 mg) and clopidogrel (300 mg). All patients who received stents were prescribed aspirin (81 mg) indefinitely. Patients who received a BMS were prescribed clopidogrel (75 mg daily) for 3 months. Patients who received DES were prescribed clopidogrel (75 mg daily) for 6 months.

Procedural details were recorded including the location of stenosis, type of stent, and stent deployment and dilation diameter. Technical success was defined as less than 30% residual stenosis on angiography. Complications, both intraprocedural and postprocedural, were recorded, as was the postprocedural medication regimen. In general, ultrasound was performed within one week of hepatic artery stent placement. It was then performed at 3 months and annually thereafter. Additional U/S studies were performed at the discretion of the liver transplant service as needed clinically. Cross sectional imaging was performed as needed or occasionally was performed for other indications. All postprocedural imaging studies were reviewed.

Primary patency was defined as the time from stent implantation to (1) an RI less than 0.5 or cross-sectional imaging showing greater than 50% narrowing; (2) a censored event (loss to follow-up, death, end of study period, re-transplant, or surgical revision); (3) hepatic artery thrombosis; and (4) any endovascular procedure required to maintain patency.

Overall survival and graft survival data were determined from the electronic health record. Overall survival was defined as the time from transplant to (1) death or (2) a censored event (loss to follow-up and end of study period). Graft survival was defined as the time from transplant to (1) re-transplant, (2) death, or (3) a censored event (loss to follow-up and end of study period). Graft survival with liver-related death was defined as the time from transplant to (1) re-transplant, (2) death (due to liver decompensation), or (3) a censored event (loss to follow-up and end of study period).

### Statistical Analyses

Numerical variables, such as age and body mass index, were summarized by mean (SD) and by median (interquartile range (IQR)). Categorical variables, such as type of liver transplant and stenosis location, were summarized by count. Survival curves were created by using the Kaplan–Meier method and compared using the log-rank test. All hypotheses tested were 2-sided, with *p* < 0.05 considered statistically significant. All analyses were performed using SAS v9.4 (SAS Institute Inc, Cary, NC, USA).

## 3. Results

### 3.1. Demographic Characteristics

During the study period, 52 patients (31 men, 21 women) with HAS were treated with stent placement. Their mean (range) age was 57 (18–69) years. Of the patients, 37 received BMS and 15 received DES. The median (range) time from transplant to intervention was 124 (19–4756) days. The median (range) RI was 0.38 (0.0–0.55). Two patients with RIs greater than 0.5 had cross-sectional imaging findings suggestive of HAS, and the decision was made to treat. Most patients were asymptomatic and were found to have hepatic artery stenosis on routine surveillance U/S. Of the 52 patients, 38 (73%) had a normal total bilirubin (<1.2 mg/dL) and 45 (87%) had a bilirubin <2.0 mg/dL (Table 1).

### 3.2. Procedural Details

The most common stenosis location was the anastomosis, and 13/52 patients had a stenosis with kinking or curvature of over 90° (Table 2). The mean stent deployment diameter (largest diameter angioplasty performed in a stent) for all patients was 4.82 mm (range, 3.0–8.0 mm). For DES, the mean diameter was 4.1 mm; for BMS, the mean diameter was 5.1 mm.

The technical success rate was 100%. Three intra-procedural complications occurred, all dissections. All three dissections were recognized at stent placement and treated with additional stents, which resulted in restoration of flow. There was one delayed complication, a pseudoaneurysm that developed just proximal to the stent 7 months after initial stent placement. The hepatic artery and pseudoaneurysm ultimately thrombosed.

### 3.3. Post Procedure

On the first post-procedure ultrasonography examination, the median (range) RI was 0.58 (0.0–0.74) from the pre-procedure RI of 0.38. Compared with baseline, 87% (45/52) of patients had higher RIs, and 10% (5/52) had lower RIs (Table 3). RI did not change in 1 patient who had a high-grade narrowing and undetectable flow. Despite successful stent placement and flow restoration, the artery thrombosed within 1 day. One patient did not have follow-up imaging because of extensive comorbid conditions.

### 3.4. Primary Patency

**Figure 1:**
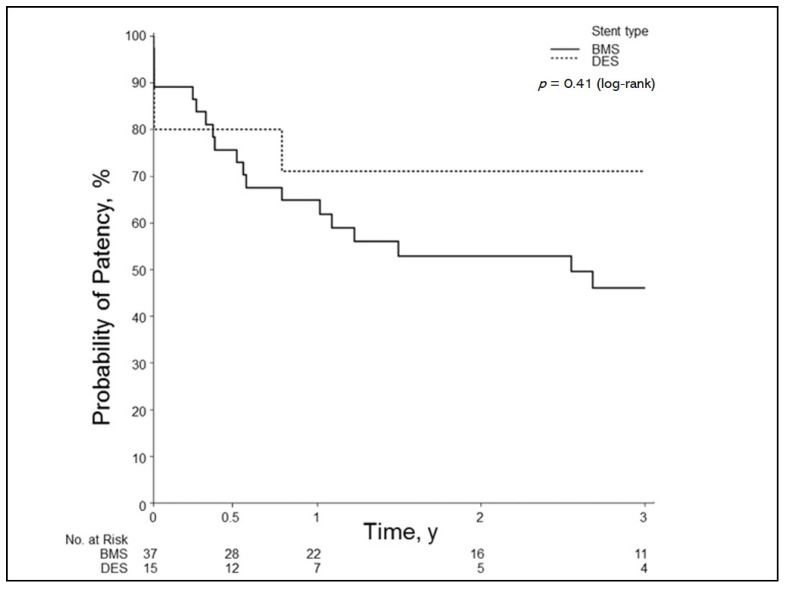
Patency outcomes by stent type for all arteries. Results of Kaplan–Meier analysis show primary patency of DES vs. BMS in hepatic artery stenosis. BMS indicates bare-metal stent; DES, drug-eluting stent. y, year(s).

The overall primary patency for all stents was as follows: 6 months, 77%; 1 year, 67%; 2 years, 56%; and 3 years, 50%. When the groups were divided into stent type, the BMS group had the following patency rates: 6 months, 76%; 1 year, 65%; 2 years, 53%; and 3 years, 46%. The DES group had patency rates at 6 months of 80%; 1 year, 71%; 2 years, 71%; and 3 years, 71%. This difference was not significant (*p* = 0.41) (Figure 1).

### 3.5. Primary Patency of Small-Caliber Arteries

**Figure 2:**
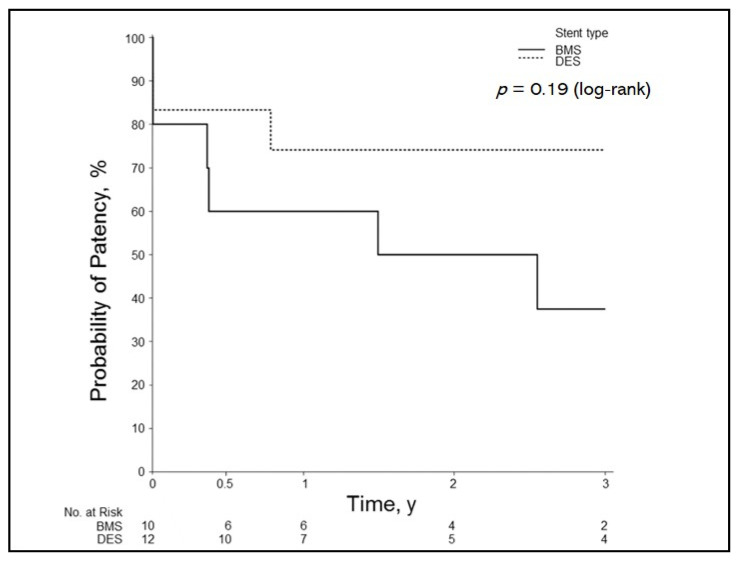
Patency outcomes by stent type for small-caliber arteries. Results of Kaplan–Meier analysis show primary patency of DES vs. BMS in hepatic artery stenosis for stent diameters of 3.5–4.5 mm. BMS indicates bare-metal stent; DES, drug-eluting stent. y, year(s).

The overall primary patency in small-caliber arteries (3.5–4.5 mm) at 6 months was 73%; 1 year, 68%; 2 years, 62%; and 3 years, 55%. When the data were divided into stent type, the BMS group had patency rates at 6 months of 60%; 1 year, 60%; 2 years, 50%; and 3 years, 38%. The DES group had patency rates at 6 months of 83%; 1 year, 74%; 2 years, 74%, and 3 years, 74%. This difference was not significant (*p* = 0.19) (Figure 2).

### 3.6. Overall Survival and Graft Survival

**Figure 3:**
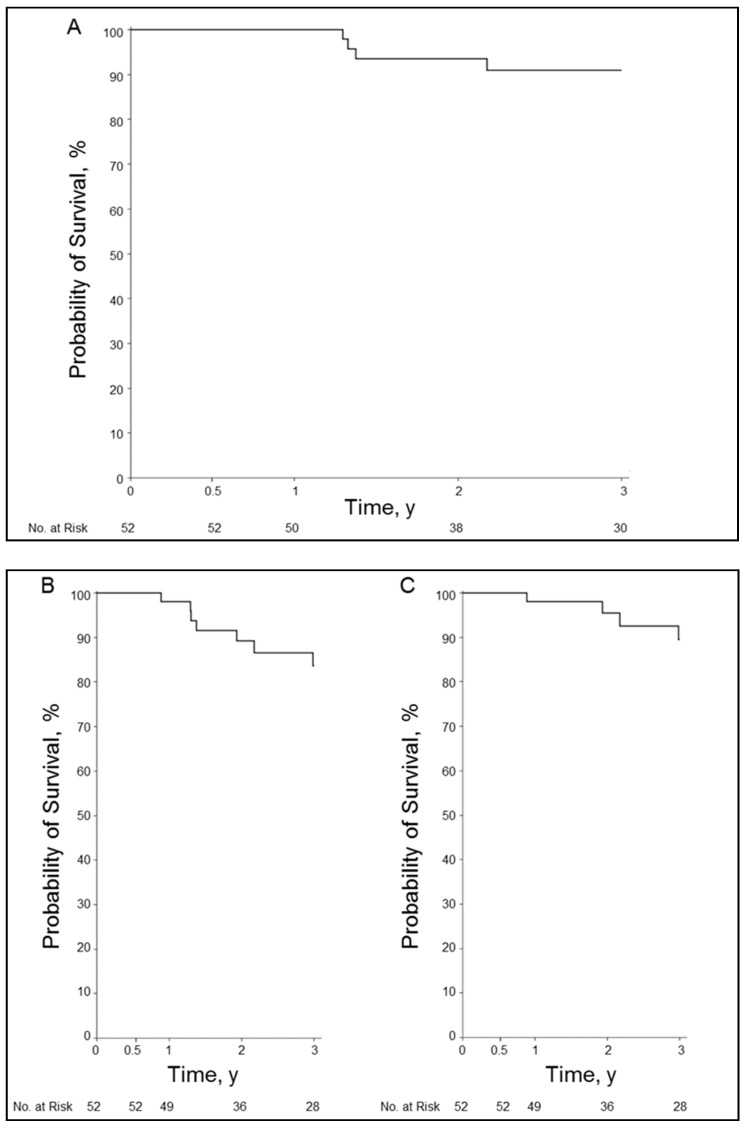
Results of stent treatment for hepatic artery stenosis. (**A**), Kaplan–Meier curve for overall survival. (**B**), Kaplan–Meier curve for graft survival. (**C**), Kaplan–Meier curve for graft survival with liver-related death. y, year(s).

Overall survival at 6 months was 100%; 1 year, 100%; 2 years, 94%; and 3 years, 91%. Graft survival at 6 months was 100%; 1 year, 98%; 2 years, 89%; and 3 years, 84%. Graft survival with liver-related death at 6 months was 100%; 1 year, 98%; 2 years, 96%; and 3 years, 90% (Figure 3A–C).

## 4. Discussion

The use of DES for HAS was first reported in a series of three patients with early onset hepatic artery thrombosis [13]. While the results were suboptimal, the indication included arterial thrombosis, a criterion that we excluded from our study. DES for liver transplant HAS was also reported in two large series [14,15]. The indication in the report by Le et al. [14] was recurrent stenosis and small-caliber arteries. However, the specific DES outcomes and the comparative outcomes of DES vs. BMS were not described.

Our study showed improved patency of DES over BMS, particularly in small-caliber arteries, although the results were not statistically significant. In reports from the coronary artery literature, comparisons of DES vs. BMS have shown that DES reduce the incidence of restenosis and target lesion revascularization [16,17]. DES have also been used in other vascular territories, including for TRAS. The small-diameter renal arteries are a risk factor for restenosis after stent placement in transplant patients [8]. A prospective study compared the use of DES in renal arteries with diameters less than 5 mm and BMS in renal arteries with diameters greater than 5 mm and showed both stents to be effective in treating TRAS but recommended BMS for the larger caliber arteries and DES for those with smaller diameters [12]. A retrospective study designed to evaluate DES, BMS, and percutaneous transluminal angioplasty in TRAS showed improved patency with DES and BMS over angioplasty alone [18]. This study also showed significantly improved patency of DES in arteries of 5 mm or less in the post-anastomotic TRAS subtype. The authors of this study suggested a tailored approach to TRAS, wherein the type of stent used depends on vessel diameter, subtype of TRAS, and other relevant anatomic factors, such as stenosis at a bifurcation. Our results also support the use of DES in smaller caliber arteries for HAS.

The overall primary patency rates in our study were similar to those of other retrospective series reporting on similar techniques and follow-up regimens. Rajakannu et al. [19] showed patency rates of 68% at 1 year and 63% at 3 years for 27 patients undergoing endovascular treatment for HAS; however, 4 of the 27 patients had angioplasty only. Sommacale et al. [7] showed 1-year patency of 65% in 37 patients, all of whom were treated with stents.

Our patency rates were lower than those from a study by Sarwar et al. [5] who reported 1-year patency for 29 patients of 90%, which remained unchanged at 10 years. A study by Le et al. [14] showed patency rates of 78% at 1 and 2 years. The differences between our results and those of these two studies may be related to the definition of patency. In the other studies, patency was time from intervention to evidence of HAS or to a censored event. We used a strict definition for loss of patency in HAS of RI less than 0.5 on surveillance Doppler ultrasonography. We believe this was a reasonable value to use because an RI less than 0.5 is generally an indication for further imaging or intervention after liver transplant [20]. However, there were patients in our series who had RIs in the 0.4–0.5 range after stent placement who were not retreated and showed no clinical evidence of liver deterioration. These vessels would have likely been considered patent in other studies but were considered as loss of patency in our study.

Generally, our practice has been to treat HAS with stent placement over angioplasty alone. Other recent studies have also shown improved patency with stent placement over angioplasty alone, as well as increased time to re-intervention in stent groups [5,14,19]. However, a meta-analysis of stent placement from 1970 to 2011 in patients with HAS did not show a significant difference in patency, complications, or requirements for intervention between angioplasty and stent placement [21], although the study was limited by its combining numerous small case series.

Previous studies [22,23] have shown that a rich collateral arterial blood supply may develop after documented stent occlusion. In our study, three patients with occlusion on cross-sectional imaging had flow in the intrahepatic arteries on ultrasonography and low/normal RIs. One of the three patients had an RI greater than 0.5. None of the three patients had clinical evidence of hepatic dysfunction or biliary ischemia. Such cases raise the question of whether all HAS cases warrant treatment. Pulitano et al. [24] retrospectively reviewed their HAS experience. They concluded that asymptomatic patients with late-onset HAS (≥6 months) do not need endovascular therapy. Currently, our practice is to attempt endovascular repair for all patients with HAS regardless of time from transplant, even if a patient is asymptomatic and has normal liver function tests. However, we agree that the time of evolution from HAS to thrombosis is critically important. In our opinion, if the onset is gradual and the liver has enough time to develop collateral blood supply, patients may not have issues with graft function related to arterial insufficiency.

In liver transplant, HAS can be caused by many factors including vessel trauma unrelated to the anastomosis, intimal hyperplasia at the anastomosis, atherosclerotic disease, or transplant immune-mediated arteriopathy [9] as a form of rejection. In immune-mediated arteriopathy, macrophage and mesenchymal cells in the intima are thought to lead to obstructive arteriopathy. DES can reduce cellular proliferation of smooth muscle cells, prevent intimal hyperplasia, and, thus, decrease restenosis.

**Figure 4:**
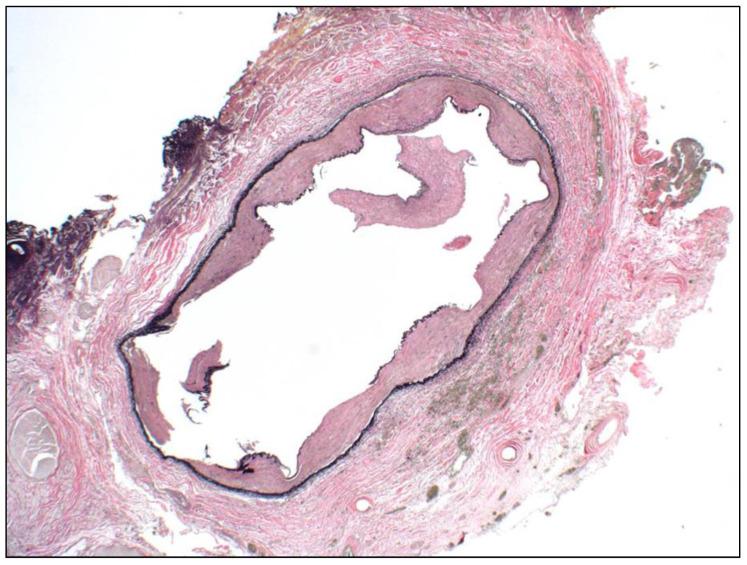
Representative photomicrograph of a resected, cross-sectioned, previously stented portion of a hepatic artery. The specimen is from the area of the previous anastomosis and is shown after electrolytic dissolution of the stent by reverse electroplating. Mild intimal atherosclerosis is present, which predates the insertion of the stent, but there is no neointima formation on the luminal side of the stent or on the surfaces of the struts. The vessel remains widely patent. Multiple indentations from the stent struts are still present in the intima, even though the stent was dissolved. Focal fragments of intimal atherosclerosis are present in the lumen, but this finding is an artifact from tissue processing. (Verhoeff–Van Gieson elastic stain, original magnification ×200).

Early in our experience, a patient who was treated with a DES for HAS underwent a surgical revision for a kink distal to the patent stent 6 months after stent implantation. The removed arterial specimen, which included the stent, was evaluated after the stent was dissolved by electrolysis [25]. The specimen showed minimal intimal fibroplasia within the intima (Figure 4).

### Limitations

The present study had several limitations. First, the study was retrospective and from a single institution, which could limit generalizing outcomes, and we evaluated outcomes over 15 years. Although 15 years enabled good long-term follow-up, endovascular techniques and devices could have changed over this period and confounded the results. There was no standardization of practice—the operator was solely responsible for choosing the stent type (BMS vs. DES), and, therefore, many types of stents were used, including different types of BMS and DES loaded with different anti-proliferative drugs. Although DES were generally used for small-caliber arteries, this was not a requirement. Additionally, the number of patients included in the study was small, which makes significance difficult to demonstrate—even though the study represents one of the larger series of patients with HAS treated with stents.

The second limitation was the use of ultrasonography for follow-up and an RI value greater than 0.5 to define patency. One patient with a known occlusion and well-developed collateral circulation had an RI greater than 0.5. Likewise, we had multiple patients with RIs in the 0.4–0.5 range who were doing well clinically. They were not treated for HAS, were followed up with ultrasonography, and showed no clinical deterioration. These patients were classified as “loss of patency.” Future studies to evaluate DES in liver transplant HAS would benefit from larger numbers, perhaps requiring a multi-center study. Ideally, a prospective randomized trial comparing the two stent types would be performed.

## 5. Conclusions

In our study, endovascular treatment of liver transplant HAS was safe and effective, and the complication rate was low. Patency rate, overall survival, and graft survival were also very good in this patient population. DES had somewhat better patency rates than BMS, and the differences were even greater favoring DES over BMS for stent diameters less than 4.5 mm, although the results were not significant. For small-caliber arteries in liver transplant HAS, treatment with DES can be considered.

## Figures and Tables

**Table 1:** Demographic and clinical characteristics of patients undergoing drug-eluting or bare-metal stent placement.

	Stent Type		
Characteristic	Drug-Eluting (*n* = 15)	Bare-Metal (*n* = 37)	Total (*n* = 52)
Age, mean (SD), y	57 (11)	57 (10)	57 (10)
Range	31–69	18–69	18–69
Women, No. (%)	6 (40)	15 (41)	21 (40)
BMI, mean (SD), kg/m^2^	23.9 (4.9)	27.4 (5.1)	26.2 (5.3)
Type of liver transplant			
Whole	13	32	45
Split	2	5	7
Laboratory values before intervention, mean (SD)			
AST	55.1 (51.6)	46.4 (37.9)	48.2 (42.0)
ALT	54.8 (55.0)	69.8 (65.6)	65.4 (62.6)
Alkaline phosphatase	282.1 (344.1)	234.5 (254.9)	248.2 (280.8)
Total bilirubin	0.97 (0.75)	1.0 (1.2)	1.0 (1.1)
Serum creatinine	1.2 (0.4)	1.4 (0.4)	1.3 (0.4)
Lowest RI before intervention, mean (SD)	0.37 (0.09)	0.36 (0.12)	0.36 (0.11)
Range	0.25–0.55	0.0–0.49	0.0–0.55

Abbreviations: ALT, alanine aminotransferase; AST, aspartate transaminase; BMI, body mass index; RI, resistive index. y, years.

**Table 2:** Procedural details of stent placement.

	Stent Type		
Variable	Drug-Eluting (*n* = 15)	Bare-Metal (*n* = 37)	Total (*n* = 52)
Days from transplant to intervention, mean (SD)	263 (427)	272 (784)	269 (696)
Median (IQR)	124 (108–208)	123 (52–136)	124 (58–138)
Range	26–1752	19–4756	19–4756
Angiographic findings			
Single stenosis	11	31	42
Tandem or multiple stenoses	4	6	10
Occlusion	0	0	0
Stenosis type			
Single straight	11	22	33
Single area of curvature, kinking ^a^	1	9	10
Multiple straight	2	4	6
Multiple areas of kinking	1	1	2
Mix of straight and kinking	0	1	1
Stenosis location			
Pre-anastomosis	0	1	1
Anastomosis	11	30	41
Post-anastomosis	0	0	0
Pre-anastomosis and anastomosis	3	4	7
Post-anastomosis and anastomosis	1	2	3
Wire system			
0.014″ (%)	15 (100)	28 (76)	43 (83)
0.035″ (%)	0 (0)	9 (24)	9 (17)
Stent type			
Balloon expandable, No. (%)	15 (100)	31 (84)	46 (89)
Self-expandable, No. (%)	0 (0)	6 (16)	6 (11)
Stent diameter, mean (SD), mm	4.1 (0.5)	5.1 (1.0)	4.8 (1.0)
Range	3.0–5.0	3.0–8.0	3.0–8.0
Technical success, No. (%)	15 (100)	37 (100)	52 (100)
Complications, No. (%)	0	4 (11)	4 (8)
Dissection, No. (%)	0	3 (75)	3 (75)
Pseudoaneurysm, No. (%)	0	1 (25)	1 (25)

Abbreviation: IQR, interquartile range. ^a^ Kinking or curvature over 90°.

**Table 3:** RIs before and after primary stent placement.

Variable	Overall (*n* = 52)
Lowest pre-intervention RI in the left or right hepatic artery	
Mean (SD)	0.36 (0.11)
Range	0.0–0.55
First post-intervention RI	
Mean (SD)	0.55 (0.14)
Range	0.0–0.74
RI change, pre-intervention to post-intervention, No. (%)	
Higher	45 (88)
Lower	5 (10)
No change	1 (2)
No follow-up study	1 (2)

Abbreviation: RI, resistive index.

## Data Availability

The data presented in this study are available on request from the corresponding author. The data are not publicly available due to identity reasons.
